# Therapeutic potential of anti-thymocyte globulin in type 1 diabetes: A systematic review

**DOI:** 10.1371/journal.pone.0323642

**Published:** 2025-05-13

**Authors:** Rahul Mittal, Keelin McKenna, Joana R. N. Lemos, Shreya Juneja, Mannat Mittal, Khemraj Hirani

**Affiliations:** 1 Diabetes Research Institute, University of Miami Miller School of Medicine, Miami, Florida, United States of America; 2 Division of Endocrinology, Diabetes, and Metabolism, Department of Medicine, University of Miami Miller School of Medicine, Miami, Florida, United States of America; 3 Herbert Wertheim College of Medicine, Florida International University, Miami, Florida, United States of America; The University of Toledo College of Medicine: The University of Toledo College of Medicine and Life Sciences, UNITED STATES OF AMERICA

## Abstract

**Background:**

Type 1 diabetes (T1D) is an autoimmune condition characterized by the destruction of insulin-producing beta cells in the pancreas. Anti-Thymocyte Globulin (ATG) has emerged as a promising immunomodulatory therapy aimed at preserving beta-cell function and altering the disease course. This systematic review synthesizes current evidence from the clinical trials evaluating the efficacy and safety of low-dose ATG in individuals with T1D.

**Methods:**

We conducted a comprehensive literature search of electronic databases, including PubMed (MEDLINE), Science Direct, Scopus, EMBASE, and ClinicalTrials.gov, to identify studies investigating ATG in T1D in accordance with the Preferred Reporting Items for Systematic Reviews and Meta-Analyses (PRISMA) criteria. The Joanna Briggs Institute (JBI) Critical Appraisal Tools for randomized clinical trials and case-control studies were used to assess the quality and evaluate the risk of bias in the eligible studies.

**Results:**

The primary outcomes assessed were preservation of C-peptide levels, glycemic control, and adverse events. Results indicated that ATG showed potential in preserving beta-cell function and improving clinical outcomes in recent-onset T1D. However, the incidence of adverse events, such as cytokine release syndrome and lymphopenia, necessitated careful monitoring and management.

**Conclusion:**

Low-dose ATG presents a promising therapeutic approach for modifying the progression of T1D. While early-phase trials demonstrate potential benefits in preserving beta-cell function, further large-scale, long-term studies are essential to establish optimal dosing regimens, long-term efficacy, and safety profiles. This review highlights the importance of continued research to fully elucidate the role of ATG in T1D management.

## Introduction

Type 1 diabetes mellitus (T1D) is a disease caused by autoimmune destruction of pancreatic beta cells, leading to a lack of insulin production and, therefore, hyperglycemia [1–6]. T1D is an extremely prevalent and highly morbid condition, imposing a substantial healthcare burden worldwide [7]. A global study performed in 2021, found there were approximately 8.4 million individuals worldwide having T1D [8]. In that year alone, around 0.5 million individuals were newly diagnosed. T1D has been found to be particularly problematic in low-income countries, where the remaining life-expectancy of a diagnosed 10-year-old is only 13 years. This is compared to a 10-year-old diagnosed in a high-income country, where the remaining life expectancy is 65 years. By 2040, it is estimated that there will be an increase in worldwide cases to around 13.5–17.4 million [8]. A majority of these cases are expected to arise in low-income and low-middle-income countries [8].

**Fig 1:**
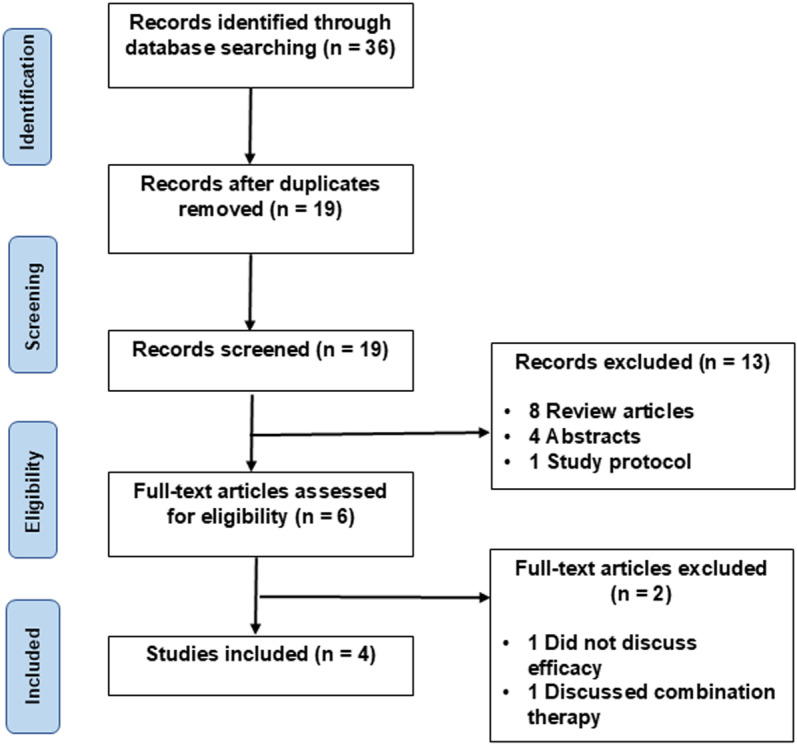
PRISMA flow diagram of study selection. This figure outlines the study selection process using the PRISMA (Preferred Reporting Items for Systematic Reviews and Meta-Analyses) framework. The flow diagram depicts the number of records identified through database searches, screening, eligibility assessments, and final inclusion in the analysis.

T1D is associated with both acute and chronic complications, with cardiovascular disease being the leading cause of death [9–12]. Acute complications include diabetic ketoacidosis (DKA), have been associated with high blood glucose levels [13,14]. Arguably more devastating, however, are the long-term effects on vasculature. These vascular complications are subdivided into micro- and macrovascular. Microvascular complications result from damage to small blood vessels and can cause retinopathy, neuropathy, and nephropathy. This damage may lead to further downstream effects, including blindness, hearing impairment, diabetic neuropathy, foot ulcers and limb amputations, and the need for hemodialysis [15–27]. Macrovascular complications result from damage to large arteries and can lead to devastating outcomes such as myocardial infarction or stroke [28–31]. Considering the high multitude of complications, studies have explored the development of novel treatment modalities for T1D such as anti-thymocyte globulin (ATG) (**Fig 1**)

T1D is thought to be a continuum, where autoantibodies are present well before the clinical manifestations of disease [32–34]. In early stages of disease, these autoantibodies are not thought to be pathogenic but instead can be useful markers of autoimmunity. Autoantibodies typically associated with T1D target insulin, 65 kDa glutamic acid decarboxylase (GAD65), insulinoma-associated protein 2 (IA-2), and zinc transporter 8 (ZNT8), with anti-insulin and anti-GAD65 being the first to develop in childhood. It has also been shown that T1D is associated with specific genotypes HLA-DR and HLA-DQ [15].

**Fig 2:**
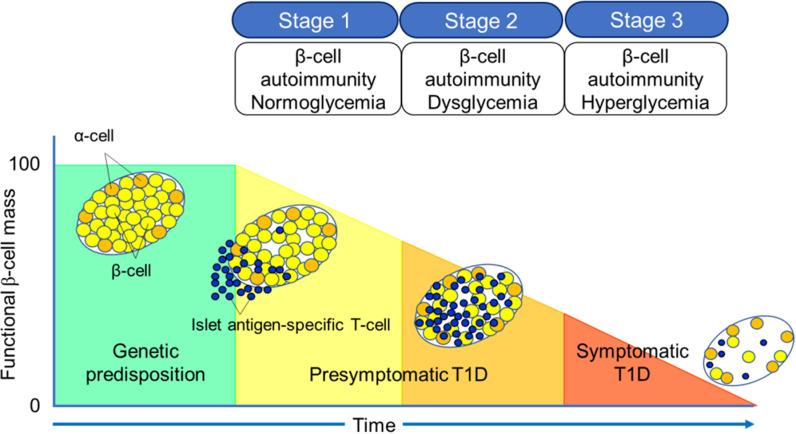
Three stages of natural history of T1D. Taken from Kawaski et al. [33] under the Creative Commons Attribution (CC BY) license.

T1D is often divided into three stages [35,36] (**Fig 2**). Stage 1 is defined as the presence of autoantibodies, without dysglycemia; stage 2 has both autoantibodies and dysglycemia; stage 3 has autoantibodies, hyperglycemia, and the clinical manifestations, including polyuria, thirst, hunger, and weight loss [15,37–40].

**Fig 3:**
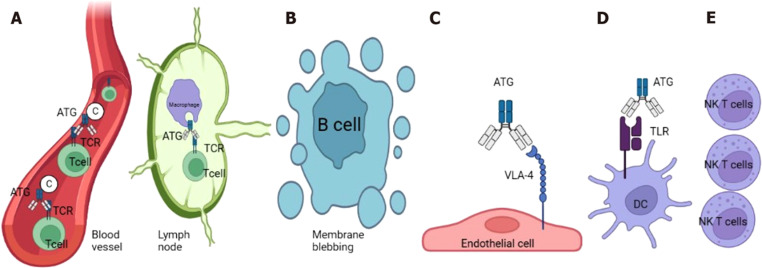
Mechanisms of action of anti-thymocyte globulin. A: T-cell depletion in blood through complement-mediated lysis and in secondary lymphoid tissue by T cell apoptosis; B: Beta-cell apoptosis by anti-thymocyte globulin (ATG); C: ATG-VLA-4 complex leading to decreased adhesion proteins in endothelial cells required by leukocyte/endothelium interaction; D: Dendritic cell maturation by HLA1/ATG interaction; E: Increased natural killer T cells. Taken from Acharya [62] under the Creative Commons Attribution Non-Commercial (CC BY-NC 4.0) license, which permits others to distribute, remix, adapt, build upon this work non-commercially, and license their derivative works on different terms, provided the original work is properly cited.

Due to the high burden of T1D with the potential ability to delay disease progression, researchers have been diligently searching for novel treatment modalities. As mentioned earlier, ATG is one of the more recently studied immunomodulators. ATG exerts its immunosuppressive effects through several mechanisms (**Fig 3**). It depletes T cells in the blood via complement-mediated lysis and induces apoptosis in secondary lymphoid tissues. ATG also induces beta-cell apoptosis and forms the ATG-VLA-4 complex, which reduces adhesion proteins necessary for leukocyte-endothelium interaction. Additionally, ATG promotes dendritic cell maturation through HLA1/ATG interaction and increases natural killer T cells, contributing to its efficacy in preventing graft-versus-host disease. These mechanisms collectively contribute to the attenuation of autoimmunity and preservation of beta-cell function, making ATG a promising therapeutic option for T1D (PMC3542267).

Beyond transplantation, ATG has demonstrated immunomodulatory potential in autoimmune conditions through both in vitro and in vivo studies. Studies have shown that ATG selectively depletes pathogenic T cells while promoting the expansion of regulatory T cells (Tregs), which play a crucial role in maintaining immune tolerance [41–43]. The preclinical T1D models treated with ATG exhibited delayed disease onset and preserved pancreatic islet function due to reduced autoreactive T-cell responses (PMC2927948). Trials using ATG for the treatment of T1D have shown an increased area under the curve (AUC) C-peptide values compared to placebo, suggesting preserved beta cell function [44–47]. Combination therapies involving ATG have also been explored extensively in vivo and translated into clinical trials. A study investigated a novel immunoregulatory strategy to prevent both alloimmune and autoimmune responses in nonobese diabetic (NOD) mice [48]. The approach combined prolonged low-dose murine anti-thymocyte globulin (mATG) with CTLA4-Ig treatment. This combination significantly delayed allograft rejection in islet transplantation models and effectively reversed established T1D in NOD mice. The treatment modulated immune responses by increasing regulatory T cells (Tregs) and reducing effector T cells (Teffs), suggesting its potential as a clinically relevant strategy for managing T1D. In agreement with the findings of this study, the combination therapy of mATG with CTLA4-Ig has been shown to significantly extended graft survival by promoting regulatory T cells (Tregs) and suppressing effector T cells (Teffs) in a fully MHC-mismatched murine skin transplant model [49]. The study also found that CTLA4-Ig inhibited the production of anti-rabbit antibodies, enhancing the efficacy of mATG. Notably, a study evaluating low-dose ATG with granulocyte colony-stimulating factor (GCSF) in T1D patients showed potential in preserving C-peptide levels and modulating immune responses [50]. However, findings indicated that ATG alone may be more effective than the combination approach, highlighting the need for further optimization of treatment regimens. These findings underscore ATG’s potential as an immunotherapeutic agent for autoimmune diseases such as T1D, warranting further investigation into its long-term efficacy and safety.

The landscape of immunotherapeutic interventions for T1D is rapidly evolving, particularly following the Food and Drug Administration (FDA) approval of Teplizumab for delaying disease onset in at-risk individuals [51–54]. This advancement highlights the necessity of systematically re-evaluating previously investigated immunotherapies, particularly those that demonstrated potential but have yet to receive regulatory approval. A critical assessment of the existing literature is essential to delineate both the therapeutic promise and the limitations of these interventions, including factors that may have influenced past trial outcomes. Such analyses are particularly valuable in identifying treatment modalities that may be more effective when administered at specific disease stages or within particular patient subpopulations. In this context, a reassessment of low-dose ATG as an immunotherapy for T1D is particularly warranted. This systematic review provides new insights into its therapeutic potential by synthesizing recent clinical evidence and addressing gaps in prior research. While previous studies have explored the immunomodulatory effects of ATG, this review uniquely focuses on its low-dose application, highlighting its ability to preserve beta-cell function and improve glycemic control while minimizing adverse effects associated with higher doses. Furthermore, immunotherapeutic strategies are likely to demonstrate greater efficacy when administered at the earliest stages of disease progression. Reevaluating the effects of low-dose ATG in specific patient populations, such as those in the preclinical or early clinical phases of T1D, has become increasingly imperative. This review also integrates novel immunological insights, such as changes in T-cell populations, which have not been comprehensively analyzed in previous studies. By critically evaluating the strengths and limitations of existing research, particularly with respect to study design, patient selection, and long-term safety, this systematic review identifies key areas requiring further investigation. It emphasizes the need for optimized dosing regimens and large-scale, long-term studies to establish the full therapeutic potential of low-dose ATG. Ultimately, this systematic synthesis advances the understanding of ATG as a viable immunotherapeutic intervention for modifying the progression of T1D and informs future research directions in the rapidly evolving field of diabetes immunotherapy.

## Methods

### Search strategy

This study adhered to the Preferred Reporting Items for Systematic Reviews and Meta-Analyses (PRISMA) guidelines and was further enhanced by following the recommendations provided in the Cochrane Collaboration Handbook. A protocol of this systematic review was designed *a priori* and was registered in the PROSPERO database (registration number: CRD4202454771). As narrative or systematic review articles were available covering studies up to 2018, searches were performed between January 1, 2018 to May 15, 2024 in the following databases: PubMed (MEDLINE), Science Direct, Scopus, and EMBASE databases using the following MeSH terms: (“Type 1 diabetes”[Mesh]) AND (“Anti-thymocyte globulin”[Mesh]); (“Type 1 diabetes”[MeSH]) AND (“Low-dose ATG”[MeSH]); (“Insulin dependent diabetes”[MeSH]) AND (“Low-dose ATG”[MeSH]) where MeSH search was not available the following Boolean terms were used (“Type 1 diabetes”) AND (“Anti-thymocyte globulin”); (“Type 1 diabetes”) AND (“Low-dose ATG”); (“Insulin dependent diabetes”) AND (“Low-dose ATG”).

### Study selection

All studies with a confirmed diagnosis of T1D in human subjects, both adults and children, who were treated with low-dose ATG (2.5 mg/kg) were included. Studies were excluded based on the following exclusion criteria: studies with no confirmed diagnosis of T1D, diabetes-induced animal models, studies that did not discuss efficacy, studies on combination treatments, review articles, meta-analyses, abstracts only, conference proceedings, editorials/letters, case reports, or articles published before January 1, 2018. Additionally, studies performed *in vitro*, or *ex vivo* were excluded. All searched titles, abstracts, and full-text articles were independently reviewed by at least two trained reviewers (K.M. and R.M.). Disagreements on inclusion and exclusion were resolved by consensus among the reviewers or through discussions with other researchers involved in this study. Articles were initially screened based on title and abstract before proceeding to full-text analysis.

### Handling of missing data

The studies included in our systematic review had no missing data relevant to the outcomes of interest. To ensure data completeness, we implemented a rigorous screening process during study selection, excluding any studies with incomplete reporting of key variables. Additionally, as part of our risk of bias assessment, we evaluated the completeness of data and study quality to ensure the reliability of our findings. This approach allowed us to maintain the integrity of our analysis without the need for imputation or other statistical methods to handle missing data.

### Data extraction

The data was extracted by at least two trained reviewers (K.M., and R.M.).

### Quality assessment

The Joanna Briggs Institute (JBI) Critical Appraisal Tools for randomized controlled trials (RCTs) and case series were used to perform quality assessment on eligible studies. For the RCTs, 13 questions were evaluated: 1) was true randomization used for assignment of participants to treatment groups?; 2) was allocation to groups concealed?; 3) were treatment groups similar at baseline?; 4) were participants blind to treatment assignment?; 5) were those delivering the treatment blind to treatment assignment?; 6) were treatment groups treated identically other than the intervention of interest?; 7) were outcome assessors blind to treatment assignment?; 8) were outcomes measured in the same way for treatment groups?; 9) were outcomes measured in a reliable way?; 10) was follow up complete and, if not, were the differences between groups adequately described?; 11) were participants analyzed in the group to which they were randomized?; 12) were appropriate statistical analyses used?; and 13) was the trial design appropriate and any deviations accounted for in the analysis? For the case series, ten questions were evaluated: 1) were there clear criteria for inclusion?; 2) was the condition measured in a standard/reliable way for all participants?; 3) were valid methods used for identification of the condition for all participants included?; 4) did the case series have consecutive inclusion of participants?; 5) did the case series have complete inclusion of participants?; 6) was there clear reporting of the demographics of the participants?; 7) was there clear reporting of clinical information of the participants?; 8) were the outcome or follow up results of cases clearly reported?; 9) was there clear reporting of the presenting site(s)/clinic(s) demographic information?; and 10) was statistical analysis appropriate? The questions were answered using “Yes”, “No”, and “Unclear”. At least two reviewers independently conducted this assessment (K.M. and R.M.) and any disagreements were resolved by consensus between the reviewers or discussion with other investigators of this study.

## Results

A total of 36 studies were retrieved using the predefined search algorithm as described in the Methods section (**Fig 1**). A total of 36 studies were retrieved using the predefined search algorithm as described in the Methods section. After removing duplicates, 19 studies were included for title and abstract screening. Following this screening, 13 studies were excluded based on irrelevance and six were included for whole-text analysis. After whole-text analysis, two articles were excluded as one did not discuss efficacy, and one discussed a combination therapy. Finally, four articles remained for inclusion in the literature review and qualitative analysis.

**Fig 4:**
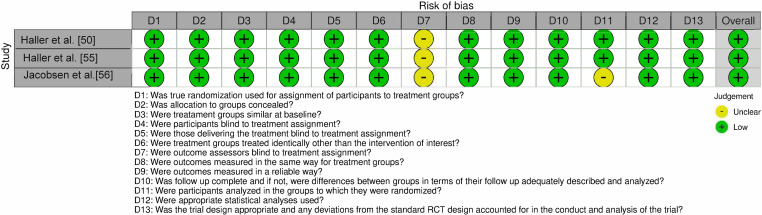
Risk of Bias (RoB) Assessment for Randomized Clinical Trials (RCTs). This figure illustrates the risk of bias evaluation across multiple domains for the included RCTs. Each domain is represented by a bar or chart segment, with color coding to indicate different levels of bias: green (low risk), and yellow (unclear risk).

**Fig 5:**
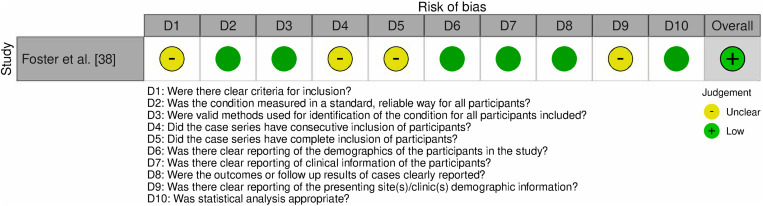
Risk of Bias (RoB) assessment for case control series. This figure illustrates the risk of bias evaluation across multiple domains for case control series. Each domain is represented by a bar or chart segment, with color coding to indicate different levels of bias: green (low risk), and yellow (unclear risk).

Across the 4 articles included, 95 human subjects were collectively evaluated [38,50,55,56]. Among these studies, the 3 RCTs included were all the same RCT analyzed at 1 and 2 years of follow up and post-hoc. These studies focused on evaluating the efficacy of treatment by monitoring key parameters such as insulin requirements, C-peptide levels, and HbA1C. One study specifically assessed immunological outcome to better understand treatment response. Among the studies, 3 were RCTs involving participants diagnosed with T1D who were randomized to receive low-dose ATG treatment, while one was a case series. The search strategy employed for the studies included in this systematic review is shown in PRISMA diagram in Fig 1. Risk of Bias for the RCT and the case series is outlined in Figs 4 and 5, respectively. It was observed that all 4 studies had a low risk of bias. In the RCTs, bias was mostly introduced due to unclear specification on whether outcome assessors were blind to treatment assignment. For the case series, bias was introduced due to lack of clear inclusion criteria for patients and lack of information on the treatment site. Overall, the studies were determined to be of appropriate quality to be included in the review. A summary of each study design, patient grouping, and included results is presented in **Table 1**.

**Table 1:** A summary of all the studies included in this systematic review.

Reference	Study	Population	Exposure	Comparison	Outcomes
Haller et al. [50], 2018	Randomized controlled trial	89 subjects aged 12–45	Low-dose ATG	Low-dose ATG/GCSF Placebo	• AUC C-peptide significantly higher in low-dose ATG vs. placebo (p = 0.0003) • ATG/GCSF vs. placebo not significantly different (p = 0.031, significance p < 0.025) • HbA1C significantly lower in both groups vs. placebo (ATG p = 0.002, ATG/GCSF p = 0.011) • No effect on insulin use • Decreased CD4 + /CD8 + ratio in both groups vs. placebo
Haller et al. [50,55], 2019	Randomized controlled trial	89 subjects aged 12–45	Low-dose ATG	Low-dose ATG/GCSF Placebo	• AUC C-peptide significantly higher in low-dose ATG vs. placebo (p = 0.00005) • ATG/GCSF vs. placebo not significantly different (p = 0.032, significance p < 0.025) • HbA1C significantly lower in both groups vs. placebo (ATG p = 0.011, ATG/GCSF p = 0.022) • No effect on insulin use • Decreased CD4 + /CD8 + ratio in both groups vs. placebo • Significant increase in Treg:Tconv ratio in both groups vs. placebo • 12-week Treg:Tconv ratio significantly associated with C-peptide (ATG p = 0.042, ATG/GCSF p = 0.028)
Jacobsen et al. [56], 2023	Randomized controlled trial	89 subjects aged 12–45	Low-dose ATG	Low-dose ATG/GCSF Placebo	• Stable methylation of *FOXP3* Treg-specific demethylation region and increased proportions of CD4 + FOXP3 + Tregs (p < 0.001) in low-dose ATG group • 2-week transient rise in IL-6, IP-10, and TNF-a (p < 0.05) • Low-dose ATG showed increased PD-1 + KLRG1 + CD57- on CD4 + cells (p = 0.011) • ATG/GCSF showed increased PD1 + CD4 + Temra MFI (p < 0.001) • Increased senescent T cells and methylation of *EOMES* correlated with decreased treatment response
Foster et al. [38], 2024	Case series	6 children aged 5–14	Low-dose ATG	None	• 50% remained at stage 2 after 18 months, 3 years, and 4 years • 50% progressed to stage 3 after 1–2 months • Those that progressed maintained good metabolic parameters • 3 children had a grade 1 cytokine release syndrome • 6 children experienced grade 3 serum sickness

Low-dose ATG is emerging as a potential treatment option to slow the progression of T1D in human subjects [38,50,55,56]. Studies have evaluated the efficacy of this treatment as discussed in the following sections:

Haller et al., [50] conducted a three-arm, randomized, double-masked, placebo-controlled phase 2b trial to better understand the effect of low-dose ATG on beta-cell function and HbA1C levels. This trial consisted of 89 subjects aged 12–45. They were randomized into three treatment groups: 29 received ATG and pegylated granulocyte colony-stimulating factor (GCSF), 29 received ATG alone, and 31 received placebo. Diphenhydramine, acetaminophen, and methylprednisolone were given pre-infusion. Subjects receiving ATG were infused with 0.5 mg/kg on the first day and 2.0 mg/kg on the second. GCSF or placebo was given subcutaneously every 2 weeks for 12 weeks. Results were analyzed at one year and the area under the curve C-peptide was found to be significantly higher in low-dose ATG alone (p = 0.0003) compared to placebo. However, the ATG/GCSF group was not significantly different from placebo (p = 0.031, significance defined as p < 0.025). HbA1C was observed to be significantly lower in both treatment groups compared to placebo (ATG p = 0.002, ATG/GCSF p = 0.011). There was no significant difference in insulin use. Finally, both treatment groups demonstrated reduced CD4 + T cells and relative preservation of CD8 + cells, resulting in a decreased CD4 + /CD8 + ratio compared to placebo. These results at one year suggest that low-dose ATG may slow decline of beta-cell function and decrease HbA1C in those with T1D.

Haller et al., [55] conducted an additional analysis of the data at two years. Results of AUC C-peptide measurements demonstrated that the low-dose ATG group was significantly higher than placebo (p = 0.00005). ATG/GCSF was not found to be significantly different (p = 0.032, significance defined as p < 0.025). HbA1C levels were found to be significantly lower in both the ATG/GCSF (p = 0.022) and ATG only (p = 0.011) groups compared to placebo. Insulin requirements were not found to be significantly different amongst the groups. Flow cytometry was also performed to understand the immunologic response to treatment. A significant reduction in the CD4 + /CD8 + ratio was noted for both treatment groups compared to placebo (p < 0.001). Absolute counts of Tconvs and Tregs were reduced in the treatment groups. However, there was a significant increase in the Treg:Tconv ratio for both ATG (2 weeks: p < 0.001, 12 weeks: p = 0.001, 24 weeks: p = 0.05) and ATG/GCSF (2 weeks: p = 0.003, 12 weeks: p = 0.004, 24 weeks: p = 0.018) compared to placebo. Mixed model analysis also showed that this change in Treg:Tconv ratio at 12 weeks was significantly associated with C-peptide (ATG p = 0.042, ATG/GCSF p = 0.028). These results are distinct from previous trials with high-dose ATG (6.5 mg/kg) that demonstrated a decreased Treg:Tconv ratio [47]. This shows that low-dose ATG, specifically, may alter the lymphocytic composition and lead to a more tolerant environment. Overall, these results suggest that low-dose ATG may be useful in the treatment of T1D, and the addition of GCSF may actually diminish the effects of ATG alone.

Jacobsen et al., [56] performed further analysis on the trial mentioned above to characterize the immunological outcomes of the treatment, which can serve as biomarkers of response to therapy. Peripheral blood from the subjects was utilized to analyze gene and protein expression, targeted gene methylation, and cytokine concentrations. It was demonstrated that treatment with low-dose ATG preserves Tregs. This was evidenced by the stable methylation of *FOXP3* Treg-specific demethylation region and increased proportions of CD4 + FOXP3 + Tregs (p < 0.001). In addition, transient rises were seen in IL-6, IL-10, and TNF-α (p < 0.05) at 2 weeks and in a CD4 + exhaustion phenotype at 12 weeks. In the low-dose ATG only group, this exhaustion phenotype was represented by an increased PD-1 + KLRG1 + CD57- on CD4 + cells (p = 0.011), while in the ATG/GCSF group, there was an increased PD1 + CD4 + Temra MFI (p < 0.001). A higher proportion of senescent T cells was seen in subjects with a less robust response to treatment that also had increased methylation of the exhaustion marker, *EOMES*. Overall, an upregulation of cytokine markers of TH1 activation, CD4 + T cell exhaustion, and neutrophil genes were found in subjects who were responding to ATG. These findings may help to identify those individuals who respond well to treatment and who may benefit from low-dose ATG or similar therapies.

Finally, Foster et al. [38] performed a case series to determine the efficacy of low-dose ATG in delaying the progression of stage 2 to stage 3 T1D. The study included six children, aged 5–14 years, who were determined to have stage 2 disease through autoantibody testing and OGTT. Prior to treatment, patients were given acetaminophen, diphenhydramine, and methylprednisolone. ATG, combined with heparin and hydrocortisone to decrease the risk of thrombophlebitis, was administered over the course of 2 days (0.5 mg/kg on day 1 and 2.0 mg/kg on day 2). These patients were followed for the next 18–48 months, and HbA1C, C-peptide, continuous glucose monitoring, insulin requirements, and side effects were evaluated. Results demonstrated that three of the six children remained at stage 2 after 18 months, 3 years, and 4 years, while three of the children progressed to stage 3 within 1–2 months after the infusion. However, those subjects were able to maintain low insulin requirements, low HbA1C, low metrics on continuous glucose monitoring, and high mixed-meal stimulated C-peptide levels. In terms of adverse reactions, three of the children experienced a grade 1 cytokine release syndrome, and all six children experienced a grade 3 serum sickness post-infusion. There were no other adverse reactions beyond the first two weeks. While this method of study is unable to produce statistically robust results, these cases suggest that low-dose ATG may be a viable option to slow progression and produce metabolically favorable profiles in patients with T1D.

## Discussion

The included studies in this systematic review suggest the potential role of low-dose ATG in modulating immune responses and preserving β-cell function T1D, contributing to a broader understanding of its therapeutic implications [38,50,55,56].. Although ATG has been associated with immunosuppressive effects and adverse events, recent studies suggest that low-dose ATG can provide a more targeted immunomodulatory effect rather than broad immune suppression [38,50,55,56]. It selectively depletes autoreactive T cells while preserving regulatory T cells (Tregs), thereby mitigating β-cell destruction [41–43]. Although ATG does not directly stimulate β-cell regeneration, it plays a clinically significant role in sustaining endogenous insulin production. Even minimal preservation of C-peptide has been linked to improved glycemic control and a reduction in long-term complications [57,58].

Despite concerns regarding long-term efficacy, studies investigating low-dose ATG have demonstrated sustained C-peptide preservation and reductions in HbA1c for at least two years [38,50,55,56]. This suggests that while ATG may not be a curative therapy, it could serve as a valuable disease-modifying agent in delaying disease progression. Additionally, while emerging therapies such as Teplizumab offer more targeted approaches, their long-term validation remains limited. Given the urgent need for effective interventions in T1D, low-dose ATG remains a viable immunotherapy, warranting further investigation to optimize dosing strategies and identify patient subgroups most likely to benefit. Furthermore, previous studies that investigated low-dose ATG in combination with GCSF provided additional evidence supporting the safety and potential efficacy of ATG in T1D [59,60]. These studies add to the overall number of individuals with T1D who have received low-dose ATG, further informing its therapeutic potential. In this context, the Minimal Effective Low Dose of Anti-Thymocyte Globulin (MELD-ATG) trial is evaluating the safety, tolerability, and efficacy of low-dose ATG in preserving β-cell function in individuals aged 5–25 years with newly diagnosed T1D [61]. It is phase II, multicenter, randomized, double-blind, placebo-controlled study conducted in Europe. The primary objective of the trial is to determine the minimal effective dose of ATG required to sustain endogenous insulin secretion, evaluated through stimulated C-peptide levels measured during a mixed meal tolerance test (MMTT) over a 12-month period. Participants are randomly assigned to different treatment arms, receiving either a placebo or one of several ATG doses (ranging from 0.1 mg/kg to 2.5 mg/kg), administered via intravenous infusion over two consecutive days. Furthermore, the trial integrates comprehensive molecular and immunological assessments to delineate the mechanisms of action underlying ATG’s immunomodulatory effects in individuals with new-onset T1D. The MELD-ATG trial is poised to generate critical data that will aid in optimizing ATG dosing regimens for the preservation of β-cell function while mitigating potential immunosuppressive risks. The findings from this study will contribute to refining the clinical utility of ATG, identifying patient subgroups most likely to derive therapeutic benefit, and advancing the landscape of disease-modifying therapies in T1D.

In considering ATG’s safety profile, cytokine release syndrome (CRS) and lymphopenia are well-recognized adverse effects, but they are dose-dependent and more commonly observed with high-dose ATG used in transplantation settings. In contrast, trials investigating low-dose ATG in T1D have reported milder, transient immune-related adverse events that are generally manageable with premedication and supportive care [38,50,55,56]. Additionally, while anti-CD3 therapies such as Teplizumab are associated with transient cytokine-related side effects that typically resolve within weeks, ATG-induced effects similarly tend to be short-lived, and its immunomodulatory benefits can extend beyond two years. Thus, rather than focusing solely on side-effect profiles, a comprehensive risk-benefit assessment is necessary to position ATG within the broader landscape of T1D immunotherapy.

Regarding the relevance of ATG compared to Teplizumab, the recent FDA approval of Teplizumab for delaying T1D progression in stage 2 patients marks a major milestone [51–54]. However, ATG operates via a distinct immunomodulatory mechanism that may offer complementary or alternative therapeutic benefits. While Teplizumab specifically targets CD3 + T cells to induce immune tolerance [51–54], ATG exerts broader immunomodulation by depleting autoreactive T cells and preserving Tregs [41–43]. Additionally, clinical trials have demonstrated that low-dose ATG preserves C-peptide levels, reduces HbA1c, and improves immune profiles, with effects lasting at least two years. Importantly, Teplizumab is currently approved for autoantibody-positive individuals at risk of developing T1D (stage 2) [51–54], whereas ATG has been studied in newly diagnosed patients (stage 3) [38,50,55,56]., suggesting that these therapies may be applicable at different stages of disease progression. Future research should explore whether ATG and Teplizumab could be used synergistically or whether specific patient subgroups may benefit more from one therapy over the other.

Although ATG is not the only immunotherapeutic option for T1D, it offers distinct advantages that justify its continued investigation. Unlike many emerging therapies that primarily target specific immune pathways, ATG provides broader immunomodulation, which may be beneficial in addressing the multifaceted immune dysregulation underlying T1D. Additionally, randomized trials have demonstrated that low-dose ATG can sustain C-peptide levels, reduce HbA1c, and improve immune profiles, with effects persisting beyond two years. While therapies such as Teplizumab have also shown promise, direct comparative studies evaluating their long-term efficacy and durability of response remain limited. Given that no single therapy has yet emerged as a definitive solution for T1D, ATG remains a viable candidate, particularly in the context of combination strategies that could further enhance its disease-modifying potential.

## Limitations

There are several limitations to these results that should be noted. Firstly, there is a scarcity of studies discussing this treatment option. Despite a thorough search, only a few studies met the inclusion criteria. The limited number of studies reduces the robustness of the conclusions and highlights the need for more extensive research in this area.

Additionally, the included studies had small sample sizes, which limits the generalizability of the findings. Small sample sizes increase the risk of statistical errors and make it difficult to draw definitive conclusions about the treatment’s impact on the broader population of individuals with T1D. Larger sample sizes in future studies would provide more reliable data and help to confirm these preliminary findings.

Moreover, while the majority of the included studies were RCTs, which are considered the gold standard in clinical research, one study was a case series. Case series are generally considered to be a lower level of evidence compared to RCTs because they lack the rigorous controls needed to establish causality. The inclusion of a case series may therefore introduce bias and affect the overall strength of the evidence.

Another significant limitation is the duration of the follow-up periods. The longest follow-up in the included studies was only four years. This relatively short duration makes it challenging to assess the long-term effects and sustainability of the treatment. Chronic conditions such as T1D require long-term studies to evaluate the enduring efficacy and potential long-term adverse effects of any therapeutic intervention.

Finally, publication bias cannot be ruled out. Studies with positive outcomes are more likely to be published, while those with negative or inconclusive results may remain unpublished. This bias could skew the overall assessment of the treatment’s efficacy and safety.

In conclusion, while the findings of this review suggest potential benefits of the treatment, the limitations highlight the need for more robust studies. Future research should focus on conducting large-scale, long-term RCTs with adequate sample sizes to provide more conclusive evidence on the efficacy and safety of this treatment option for T1D.

## Conclusions and future directions

Low-dose ATG is being considered a potential treatment modality for patients with T1D. The results from this systematic review suggest that it may be utilized to slow disease progression, increase C-peptide levels, decrease HbA1C levels, and produce a favorable immunologic response. It is essential that further RCTs are conducted to better understand the use of low-dose ATG in treating T1D.

It should be noted that this treatment is not without some adverse effects. Many patients included in these studies experienced post-transfusion reactions, including serum sickness and cytokine release syndrome, lymphopenia, nausea, and rash. These reactions were easily treated with oral prednisone. Although no long-term side effects were noted, providers should be aware of these potential complications so they may be treated appropriately.

It is also imperative that the underlying mechanisms of this treatment option are better understood. These studies show that there is a change in immune cells and cytokine expression. Understanding these mechanisms will not only allow for refinement of the treatment but may also provide important information on treatment response. This would allow providers to recognize patients that may benefit from further treatment and those that are not exhibiting an appropriate response.

T1D is a highly morbid condition, causing a significant burden to the patient, as well as the healthcare system as a whole. Further research on this rapidly emerging treatment option is essential to slow disease progression, thereby decreasing disease complications and increasing patient quality of life.

## Supporting information

S1 File PRISMA checklist. (PDF)

S2 File A comprehensive spreadsheet documenting each article collected for this manuscript. (XLSX)

## References

[1] Atkinson MA, Eisenbarth GS, Michels AW. Type 1 diabetes. Lancet. 2014;383(9911):69–82. doi: 10.1016/S0140-6736(13)60591-7 23890997 PMC4380133

[2] DiMeglio LA, Evans-Molina C, Oram RA. Type 1 diabetes. Lancet. 2018;391(10138):2449–62. doi: 10.1016/S0140-6736(18)31320-5 29916386 PMC6661119

[3] Herold KC, Delong T, Perdigoto AL, Biru N, Brusko TM, Walker LSK. The immunology of type 1 diabetes. Nat Rev Immunol. 2024;24(6):435–51. 38308004 10.1038/s41577-023-00985-4 PMC7616056

[4] Ilonen J, Lempainen J, Veijola R. The heterogeneous pathogenesis of type 1 diabetes mellitus. Nat Rev Endocrinol. 2019;15(11):635–50. doi: 10.1038/s41574-019-0254-y 31534209

[5] Mittal R, Camick N, Lemos JRN, Hirani K. Gene-environment interaction in the pathophysiology of type 1 diabetes. Front Endocrinol (Lausanne). 2024;15:1335435. doi: 10.3389/fendo.2024.1335435 38344660 PMC10858453

[6] Subramanian S, Khan F, Hirsch IB. New advances in type 1 diabetes. BMJ. 2024;384:e075681. doi: 10.1136/bmj-2023-075681 38278529

[7] Schmidt AM. Highlighting diabetes mellitus: the epidemic continues. Arterioscler Thromb Vasc Biol. 2018;38(1):e1–8. 10.1161/ATVBAHA.117.310221 PMC5776687 29282247

[8] Gregory GA, Robinson TIG, Linklater SE, Wang F, Colagiuri S, de Beaufort C, et al. Global incidence, prevalence, and mortality of type 1 diabetes in 2021 with projection to 2040: a modelling study. Lancet Diabetes Endocrinol. 2022;10(10):741–60. doi: 10.1016/S2213-8587(22)00218-2 36113507

[9] Julián MT, Pérez-Montes de Oca A, Julve J, Alonso N. The double burden: type 1 diabetes and heart failure-a comprehensive review. Cardiovasc Diabetol. 2024;23(1):65. doi: 10.1186/s12933-024-02136-y 38347569 PMC10863220

[10] Maahs DM, West NA, Lawrence JM, Mayer-Davis EJ. Epidemiology of type 1 diabetes. Endocrinol Metab Clin North Am. 2010;39(3):481–97. doi: 10.1016/j.ecl.2010.05.011 20723815 PMC2925303

[11] Vergès B. Cardiovascular disease in type 1 diabetes, an underestimated danger: Epidemiological and pathophysiological data. Atherosclerosis. 2024;394:117158. doi: 10.1016/j.atherosclerosis.2023.06.005 37369617

[12] Zhen XM, Twigg SM, Wu T, Tabet E, McGill MJ, Constantino M, et al. Diabetic ketoacidosis in an adult with beta-ketothiolase deficiency (BKD) involving a novel ACAT1 variant : first report of established diabetes in BKD and a review of the literature. Clin Diabetes Endocrinol. 2024;10(1):17. doi: 10.1186/s40842-024-00174-9 38853254 PMC11163784

[13] Calimag APP, Chlebek S, Lerma EV, Chaiban JT. Diabetic ketoacidosis. Dis Mon. 2023;69(3):101418. doi: 10.1016/j.disamonth.2022.101418 35577617

[14] Dhatariya KK, Glaser NS, Codner E, Umpierrez GE. Diabetic ketoacidosis. Nat Rev Dis Primers. 2020;6(1):40. doi: 10.1038/s41572-020-0165-1 32409703

[15] Katsarou A, Gudbjörnsdottir S, Rawshani A, Dabelea D, Bonifacio E, Anderson BJ, et al. Type 1 diabetes mellitus. Nat Rev Dis Primers. 2017;3:17016. doi: 10.1038/nrdp.2017.16 28358037

[16] Melendez-Ramirez LY, Richards RJ, Cefalu WT. Complications of type 1 diabetes. Endocrinol Metab Clin North Am. 2010;39(3):625–40. doi: 10.1016/j.ecl.2010.05.009 20723824

[17] Papadopoulou-Marketou N, Chrousos GP, Kanaka-Gantenbein C. Diabetic nephropathy in type 1 diabetes: a review of early natural history, pathogenesis, and diagnosis. Diabetes Metab Res Rev. 2017;33(2):10.1002/dmrr.2841. doi: 10.1002/dmrr.2841 27457509

[18] Syed FZ. Type 1 Diabetes Mellitus. Ann Intern Med. 2022;175(3):ITC33–48. doi: 10.7326/AITC202203150 35254878

[19] Huang H, Fan Y, Yan F, Hu Y, He H, Xu T, et al. Diabetes and long duration leading to speech-, low/mid-, and high- frequency hearing loss: current evidence from the China National Health Survey 2023. J Endocrinol Invest. 2025;48(1):233–43. doi: 10.1007/s40618-024-02406-2 38869778 PMC11729146

[20] Mishra UP, Behera G, Sahoo AK, Mishra S, Patnaik R. The impact of diabetes mellitus on sensorineural hearing loss: a cross-sectional study in Eastern India. Cureus. 2024;16(1):e52431. doi: 10.7759/cureus.52431 38371046 PMC10869999

[21] Mittal R, Keith G, Lacey M, Lemos JRN, Mittal J, Assayed A, et al. Diabetes mellitus, hearing loss, and therapeutic interventions: a systematic review of insights from preclinical animal models. PLoS One. 2024;19(7):e0305617. doi: 10.1371/journal.pone.0305617 38985787 PMC11236185

[22] Mittal R, McKenna K, Keith G, Lemos JRN, Mittal J, Hirani K. A systematic review of the association of Type I diabetes with sensorineural hearing loss. PLoS One. 2024;19(2):e0298457. doi: 10.1371/journal.pone.0298457 38335215 PMC10857576

[23] Pan J-Y, Chen Y, Lin Z-H, Lv B, Chen L, Feng S-Y. Association between triglyceride-glucose index and hearing threshold shifts of adults in the united states: National health and nutrition examination survey, 2015-2016. J Multidiscip Healthc. 2024;17:1791–801. doi: 10.2147/JMDH.S454678 38686130 PMC11056606

[24] Williams ED, Rubio ME. Associations between diabetes mellitus and sensorineural hearing loss from humans and animal studies. Hear Res. 2024;450:109072. doi: 10.1016/j.heares.2024.109072 38936171 PMC12175331

[25] Mittal R, McKenna K, Keith G, McKenna E, Sinha R, Lemos JRN, et al. Systematic review of translational insights: neuromodulation in animal models for diabetic peripheral neuropathy. PLoS One. 2024;19(8):e0308556. doi: 10.1371/journal.pone.0308556 39116099 PMC11309513

[26] Mohammed MM, Shaik AM, Syeda ZR, Khare R, Bukka S, Devani A, et al. Prevalence and severity of sensorineural hearing loss in diabetic and hypertensive patients: a comparative cross-sectional study. Cureus. 2024;16(6):e62573. doi: 10.7759/cureus.62573 39027778 PMC11255532

[27] Packer C, Ali S, Manna B. Diabetic foot ulcer. Treasure Island: StatPearls Publishing; 2025. 29763062

[28] Bebu I, Schade D, Braffett B, Kosiborod M, Lopes-Virella M, Soliman EZ, et al. Risk factors for first and subsequent CVD events in Type 1 diabetes: The DCCT/EDIC study. Diabetes Care. 2020;43(4):867–74. doi: 10.2337/dc19-2292 32001614 PMC7085803

[29] Forbes JM, Cooper ME. Mechanisms of diabetic complications. Physiol Rev. 2013;93(1):137–88. doi: 10.1152/physrev.00045.2011 23303908

[30] Kerola AM, Juonala M, Palomäki A, Semb AG, Rautava P, Kytö V. Case fatality of patients with type 1 diabetes after myocardial infarction. Diabetes Care. 2022;45(7):1657–65. doi: 10.2337/dc22-0042 35679070 PMC9274223

[31] Rawshani A, Rawshani A, Franzén S, Eliasson B, Svensson A-M, Miftaraj M, et al. Mortality and cardiovascular disease in Type 1 and Type 2 diabetes. N Engl J Med. 2017;376(15):1407–18. doi: 10.1056/NEJMoa1608664 28402770

[32] ElSayed NA, Aleppo G, Aroda VR, Bannuru RR, Brown FM, Bruemmer D, et al. 2. Classification and diagnosis of diabetes: standards of care in diabetes-2023. Diabetes Care. 2023;46(Suppl 1):S19–40. doi: 10.2337/dc23-S002 36507649 PMC9810477

[33] Kawasaki E. Anti-Islet autoantibodies in Type 1 diabetes. Int J Mol Sci. 2023;24(12):10012. doi: 10.3390/ijms241210012 37373160 PMC10298549

[34] Kawasaki E, Yasui J-I, Tsurumaru M, Takashima H, Ikeoka T, Mori F, et al. Sequential elevation of autoantibodies to thyroglobulin and glutamic acid decarboxylase in type 1 diabetes. World J Diabetes. 2013;4(5):227–30. doi: 10.4239/wjd.v4.i5.227 24147207 PMC3797888

[35] Insel RA, Dunne JL, Atkinson MA, Chiang JL, Dabelea D, Gottlieb PA, et al. Staging presymptomatic type 1 diabetes: a scientific statement of JDRF, the Endocrine Society, and the American Diabetes Association. Diabetes Care. 2015;38(10):1964–74. doi: 10.2337/dc15-1419 26404926 PMC5321245

[36] Sundheim B, Hirani K, Blaschke M, Lemos JRN, Mittal R. Pre-type 1 diabetes in adolescents and teens: screening, nutritional interventions, beta-cell preservation, and psychosocial impacts. J Clin Med. 2025;14(2):383. doi: 10.3390/jcm14020383 39860389 PMC11765808

[37] Felton JL, Redondo MJ, Oram RA, Speake C, Long SA, Onengut-Gumuscu S, et al. Islet autoantibodies as precision diagnostic tools to characterize heterogeneity in type 1 diabetes: a systematic review. Commun Med (Lond). 2024;4(1):66. doi: 10.1038/s43856-024-00478-y 38582818 PMC10998887

[38] Foster TP, Jacobsen LM, Bruggeman B, Salmon C, Hosford J, Chen A, et al. Low-dose antithymocyte globulin: a pragmatic approach to treating stage 2 type 1 diabetes. Diabetes Care. 2024;47(2):285–9. doi: 10.2337/dc23-1750 38117469 PMC10834389

[39] Jacobsen LM, Larsson HE, Tamura RN, Vehik K, Clasen J, Sosenko J, et al. Predicting progression to type 1 diabetes from ages 3 to 6 in islet autoantibody positive TEDDY children. Pediatr Diabetes. 2019;20(3):263–70. doi: 10.1111/pedi.12812 30628751 PMC6456374

[40] Ziegler AG, Rewers M, Simell O, Simell T, Lempainen J, Steck A, et al. Seroconversion to multiple islet autoantibodies and risk of progression to diabetes in children. JAMA. 2013;309(23):2473–9. doi: 10.1001/jama.2013.6285 23780460 PMC4878912

[41] Boenisch O, Lopez M, Elyaman W, Magee CN, Ahmad U, Najafian N. Ex vivo expansion of human Tregs by rabbit ATG is dependent on intact STAT3-signaling in CD4⁺ T cells and requires the presence of monocytes. Am J Transplant. 2012;12(4):856–66. doi: 10.1111/j.1600-6143.2011.03978.x 22390202 PMC3777828

[42] Copic D, Direder M, Klas K, Bormann D, Laggner M, Ankersmit HJ, et al. Antithymocyte Globulin Inhibits CD8+ T Cell Effector Functions via the Paracrine Induction of PDL-1 on Monocytes. Cells. 2023;12(3):382. doi: 10.3390/cells12030382 36766722 PMC9913606

[43] Chung DT, Korn T, Richard J, Ruzek M, Kohm AP, Miller S, et al. Anti-thymocyte globulin (ATG) prevents autoimmune encephalomyelitis by expanding myelin antigen-specific Foxp3+ regulatory T cells. Int Immunol. 2007;19(8):1003–10. doi: 10.1093/intimm/dxm078 17698561

[44] Luo X, Herold KC, Miller SD. Immunotherapy of type 1 diabetes: where are we and where should we be going?. Immunity. 2010;32(4):488–99. doi: 10.1016/j.immuni.2010.04.002 20412759 PMC2860878

[45] Mohty M. Mechanisms of action of antithymocyte globulin: T-cell depletion and beyond. Leukemia. 2007;21(7):1387–94. doi: 10.1038/sj.leu.2404683 17410187

[46] Xin GLL, Khee YP, Ying TY, Chellian J, Gupta G, Kunnath AP, et al. Current status on immunological therapies for type 1 diabetes mellitus. Curr Diab Rep. 2019;19(5):22. doi: 10.1007/s11892-019-1144-3 30905013

[47] Gitelman SE, Gottlieb PA, Felner EI, Willi SM, Fisher LK, Moran A, et al. Antithymocyte globulin therapy for patients with recent-onset type 1 diabetes: 2 year results of a randomised trial. Diabetologia. 2016;59(6):1153–61. Epub 20160406. doi: 10.1007/s00125-016-3917-4 27053235 PMC4869699

[48] Vergani A, D’Addio F, Jurewicz M, Petrelli A, Watanabe T, Liu K, et al. A novel clinically relevant strategy to abrogate autoimmunity and regulate alloimmunity in NOD mice. Diabetes. 2010;59(9):2253–64. doi: 10.2337/db09-1264 20805386 PMC2927948

[49] D’Addio F, Boenisch O, Magee CN, Yeung MY, Yuan X, Mfarrej B, et al. Prolonged, low-dose anti-thymocyte globulin, combined with CTLA4-Ig, promotes engraftment in a stringent transplant model. PLoS One. 2013;8(1):e53797. doi: 10.1371/journal.pone.0053797 23326509 PMC3542267

[50] Haller MJ, Schatz DA, Skyler JS, Krischer JP, Bundy BN, Miller JL, et al. Low-Dose Anti-Thymocyte Globulin (ATG) preserves β-cell function and improves HbA1c in new-onset type 1 diabetes. Diabetes Care. 2018;41(9):1917–25. doi: 10.2337/dc18-0494 30012675 PMC6105329

[51] Herold KC, Gitelman SE, Ehlers MR, Gottlieb PA, Greenbaum CJ, Hagopian W, et al. Teplizumab (anti-CD3 mAb) treatment preserves C-peptide responses in patients with new-onset type 1 diabetes in a randomized controlled trial: metabolic and immunologic features at baseline identify a subgroup of responders. Diabetes. 2013;62(11):3766–74. doi: 10.2337/db13-0345 23835333 PMC3806618

[52] Hagopian W, Ferry RJ Jr, Sherry N, Carlin D, Bonvini E, Johnson S, et al. Teplizumab preserves C-peptide in recent-onset type 1 diabetes: two-year results from the randomized, placebo-controlled Protégé trial. Diabetes. 2013;62(11):3901–8. doi: 10.2337/db13-0236 23801579 PMC3806608

[53] Herold KC, Bundy BN, Long SA, Bluestone JA, DiMeglio LA, Dufort MJ, et al. An anti-CD3 antibody, teplizumab, in relatives at risk for Type 1 diabetes. N Engl J Med. 2019;381(7):603–13. doi: 10.1056/NEJMoa1902226 31180194 PMC6776880

[54] Ramos EL, Dayan CM, Chatenoud L, Sumnik Z, Simmons KM, Szypowska A, et al. Teplizumab and β-cell function in newly diagnosed type 1 diabetes. N Engl J Med. 2023;389(23):2151–61. doi: 10.1056/NEJMoa2308743 37861217

[55] Haller MJ, Long SA, Blanchfield JL, Schatz DA, Skyler JS, Krischer JP, et al. Low-dose anti-thymocyte globulin preserves C-peptide, reduces HbA1c, and increases regulatory to conventional T-cell ratios in new-onset Type 1 diabetes: two-year clinical trial data. Diabetes. 2019;68(6):1267–76. doi: 10.2337/db19-0057 30967424 PMC6610026

[56] Jacobsen LM, Diggins K, Blanchfield L, McNichols J, Perry DJ, Brant J, et al. Responders to low-dose ATG induce CD4+ T cell exhaustion in type 1 diabetes. JCI Insight. 2023;8(16):e161812. doi: 10.1172/jci.insight.161812 37432736 PMC10543726

[57] Lachin JM, McGee P, Palmer JP, DCCT/EDIC Research Group. Impact of C-peptide preservation on metabolic and clinical outcomes in the diabetes control and complications trial. Diabetes. 2014;63(2):739–48. doi: 10.2337/db13-0881 24089509 PMC3900540

[58] Rickels MR, Evans-Molina C, Bahnson HT, Ylescupidez A, Nadeau KJ, Hao W, et al. High residual C-peptide likely contributes to glycemic control in type 1 diabetes. J Clin Invest. 2020;130(4):1850–62. doi: 10.1172/JCI134057 31895699 PMC7108933

[59] Haller MJ, Gitelman SE, Gottlieb PA, Michels AW, Rosenthal SM, Shuster JJ, et al. Anti-thymocyte globulin/G-CSF treatment preserves β cell function in patients with established type 1 diabetes. J Clin Invest. 2015;125(1):448–55. doi: 10.1172/JCI78492 25500887 PMC4382237

[60] Haller MJ, Gitelman SE, Gottlieb PA, Michels AW, Perry DJ, Schultz AR, et al. Antithymocyte globulin plus G-CSF combination therapy leads to sustained immunomodulatory and metabolic effects in a subset of responders with established Type 1 diabetes. Diabetes. 2016;65(12):3765–75. doi: 10.2337/db16-0823 27669730 PMC5127248

[61] Wilhelm-Benartzi CS, Miller SE, Bruggraber S, Picton D, Wilson M, Gatley K, et al. Study protocol: Minimum effective low dose: anti-human thymocyte globulin (MELD-ATG): phase II, dose ranging, efficacy study of antithymocyte globulin (ATG) within 6 weeks of diagnosis of type 1 diabetes. BMJ Open. 2021;11(12):e053669. doi: 10.1136/bmjopen-2021-053669 34876434 PMC8655536

[62] Acharya S, Lama S, Kanigicherla DA. Anti-thymocyte globulin for treatment of T-cell-mediated allograft rejection. World J Transplant. 2023;13(6):299–308. doi: 10.5500/wjt.v13.i6.299 38174145 PMC10758678

